# Comparative efficacy of five most common traditional Chinese medicine monomers for promoting recovery of motor function in rats with blunt spinal cord injury: a network meta-analysis

**DOI:** 10.3389/fneur.2023.1165076

**Published:** 2023-07-03

**Authors:** Luchun Xu, Yongdong Yang, Wenqing Zhong, Wenhao Li, Chen Liu, Ziwei Guo, Xing Yu

**Affiliations:** Department of Orthopedics, Dongzhimen Hospital, Beijing University of Chinese Medicine, Beijing, China

**Keywords:** animal studies, traditional Chinese medicine, monomer, spinal cord injury, motor function, network meta-analysis

## Abstract

**Objective:**

This research employed a network meta-analysis (NMA) to examine the effectiveness of five traditional Chinese medicine (TCM) monomers for promoting motor function recovery in rats with blunt spinal cord injury (SCI).

**Methods:**

Wangfang, China National Knowledge Infrastructure, Web of Science, Embase, Chinese Scientific Journal Database, PubMed, and the Chinese Biomedical Literature Databases were searched for retrieving relevant articles published from their inception to December 2022. Two reviewers performed screening of search results, data extraction, and literature quality assessment independently.

**Results:**

For this meta-analysis, 59 publications were included. Based on the recovery of motor function at weeks 1, 2, 3, and 4 in NMA, almost all TCM groups had significantly increased positive effects than the negative control animals. In terms of cumulative probability, the tanshinone IIA (TIIA) group ranked first in restoring motor function in the first week after blunt SCI, and the resveratrol (RSV) group ranked first during the last 3 weeks.

**Conclusion:**

The NMA revealed that TCM monomers could effectively restore motor function in the rat model of blunt SCI. In rats with blunt SCI, TIIA may be the most effective TCM monomer during the first week, whereas RSV may be the most effective TCM monomer during the last 3 weeks in promoting motor function recovery. For better evidence reliability in preclinical investigations and safer extrapolation of those findings into clinical settings, further research standardizing the implementation and reporting of animal experiments is required.

**Systematic Review Registration:**

https://inplasy.com/, identifier INPLASY202310070.

## 1. Introduction

Spinal cord injury (SCI) is a kind of disease that affects the central nervous system and is linked to a significant risk of disability and death. In addition, the incidence of SCI is increasing (1–3). Of all the factors that contribute to SCI, trauma is the most common causal factor observed in clinical cases. SCI can broadly be categorized into two injuries: primary and secondary. Primary injury is caused by direct external force on the spinal cord during the trauma. After the primary injury, the activity of factors such as inflammation, oxidative stress, autophagy, and apoptosis gradually expands across the spinal tissue, causing secondary injuries. Such injuries cause extensive and sustained damage, leading to permanent loss of motor and sensory function (4–6). The current treatment options for SCI include decompression surgery, medication, and physical therapy; irrespective, there are no satisfactory treatments for SCI (7, 8). As a result, the treatment of SCI remains a major challenge. Many injury models have been used in the study of SCI, among which these blunt injury models, where the spinal cord is compressed or contused, imitate common human injuries and offer an excellent setting for research into secondary pathophysiological processes that take place immediately following injury in a more precise manner (9, 10). Furthermore, in experimental animals, the pathophysiology of SCI is considerably similar in rats and humans (11). Rats are also less expensive and more standardized than other animal species, making them the most commonly used model for studying SCI (12, 13).

In the quest to explore effective treatments for blunt SCI, a few studies have demonstrated the neuroprotective role of traditional Chinese medicine (TCM) monomers (14, 15). At present, more than ten TCM monomers are available for the treatment of blunt SCI, five of which have been extensively studied: curcumin (CUR), tetramethylpyrazine (TMP), resveratrol (RSV), ginsenoside (GS), and tanshinone IIA (TIIA). CUR is a hydrophobic polyphenol that is the biologically active component of turmeric (16, 17). TMP is a monomer alkaloid that is extracted from the dried rhizome of *Ligusticum chuanxiong*, a Chinese herbal medicine (18, 19). RSV is a polyphenol found in berries and wine that is used in TCM (20, 21). GSs are steroid glycosides and triterpene saponins obtained from *Panax ginseng*, a plant that has been historically used for medicinal purposes (22, 23). Almost all GSs have anti-inflammatory and antioxidant properties and thus their medicinal potential is often analyzed together (24). TIIA is a monomer obtained from the liposoluble extract of *Salvia miltiorrhiza* Bunge (25, 26). Animal studies have demonstrated that these five TCM monomers assume a neuroprotective role in blunt SCI through several mechanisms to promote the recovery of motor function. Irrespective, it is still unclear which of these monomers is the most suitable for motor function recovery after blunt SCI. Therefore, the present study aimed to assess data from rat studies on the use of TCM monomers for treating blunt SCI. Subsequently, a network meta-analysis (NMA) was employed to examine the effectiveness of five TCM monomers for promoting motor function recovery in rats with blunt (SCI). The results obtained will provide a theoretical basis for the treatment of blunt SCI using TCM monomers and lay a foundation for future research, which can promote the use of animal data in clinical research.

## 2. Materials and methods

### 2.1. Registration

The guidelines outlined in the Preferred Reporting Items for Systematic Reviews and Meta-Analyses were followed throughout this investigation (27). The research protocol was submitted to INPLASY for registration (registration number: INPLASY202310070).

### 2.2. Search strategy and selection criteria

The following databases were searched: Chinese Biomedical Literature, Wanfang, Chinese Scientific Journal, China National Knowledge Infrastructure, Web of Science, Ovid-Embase, and PubMed (inception to December 2022). Potentially eligible papers were identified using the following terms as topic words, keywords, free-text terms, or Medical Subject Heading terms: “curcumin,” “turmeric yellow,” “curcumin phytosome,” “ginsenoside,” “panaxosides,” “resveratrol,” “trans-resveratrol,” “cis-resveratrol,” “tanshinone,” “tanshinone IIA,” “tetramethylpyrazine,” “ligustrazine,” “chuanxiongzine,” “spinal cord injuries,” “spinal cord injury,” “spinal injury,” “spinal cord trauma,” and “spinal cord contusion.” An individualized database-specific approach was used for each search. Methods of blinding, languages used, and publishing dates were unrestricted. The detailed search strategy of each database is provided in Supplementary Table S1.

All enrolled studies followed the criteria: (1) Animals: rat with blunt SCI (contusion and compression model); (2) Intervention: five TCM monomers (CUR, TMP, RSV, GS, and TIIA); (3) control: placebo or no treatment; (4) Outcome: Weekly Basso-Beattie-Bresnahan (BBB) Locomotor Rating scale scores over 4 weeks. (5) Study type: control studies. Studies were excluded if (1) SCI was induced by other means, such as complete transverse SCI, hemisection SCI, and spinal cord ischemia/reperfusion injury; (2) BBB scores were not reported; (3) studies were duplicated; and (4) if complete raw data were not provided or if data could not be extracted.

### 2.3. Data collection and quality assessment

Two trained researchers were involved in selecting articles and extracting data from eligible studies in full compliance with the criteria for inclusion/exclusion as well as in cross-checking them. Disagreements were resolved by a third researcher. Relevant data were extracted according to a standard checklist, which included two major parameters: basic data (author [s], publication year, country, study design; species, age, sex, weight, size of the sample, animal modeling methods, type of TCM monomer, dose, and route) and outcomes (BBB score).

Using SYRCLE’s Risk of Bias tool for animal research, the reviewers conducted independent assessments of the quality of the articles that were included in the analysis (28). The following ten criteria were used to assess possible bias in the enrolled studies: (1) sequence generation, (2) baseline characteristics, (3) allocation concealment, (4) random housing, (5) blinded animal intervention, (6) random outcome assessment, (7) blinded outcome assessment, (8) incomplete outcome data, (9) selective outcome reporting, and (10) other types of bias. A third reviewer was consulted to settle any disagreements of opinion that may have arisen. Each study was graded to either be of “low,” “high,” or “unclear” risk.

### 2.4. Statistical analysis

All variables included in our study were continuous variables. These variables were retrieved for NMA using STATA version 16, and the standardized mean difference (SMD) and the respective 95% confidence interval (CI) were calculated. Subsequently, the evidence network diagram was created to compare the five TCM monomers and allow a visual assessment of the relationship between each monomer and sample size. The thickness of the line segment indicated the number of studies that examined both interventions, whereas the size of the circles indicated the sample sizes used. Various intervention probabilities were ranked using SUCRA (surface under the cumulative ranking area). The SUCRA scores varied from 0 to 100%, reflecting the gradual increase from the poorest to the best treatments (29). Publication bias and small-sample effects were assessed using funnel plots.

## 3. Results

### 3.1. Literature search

**Figure 1:**
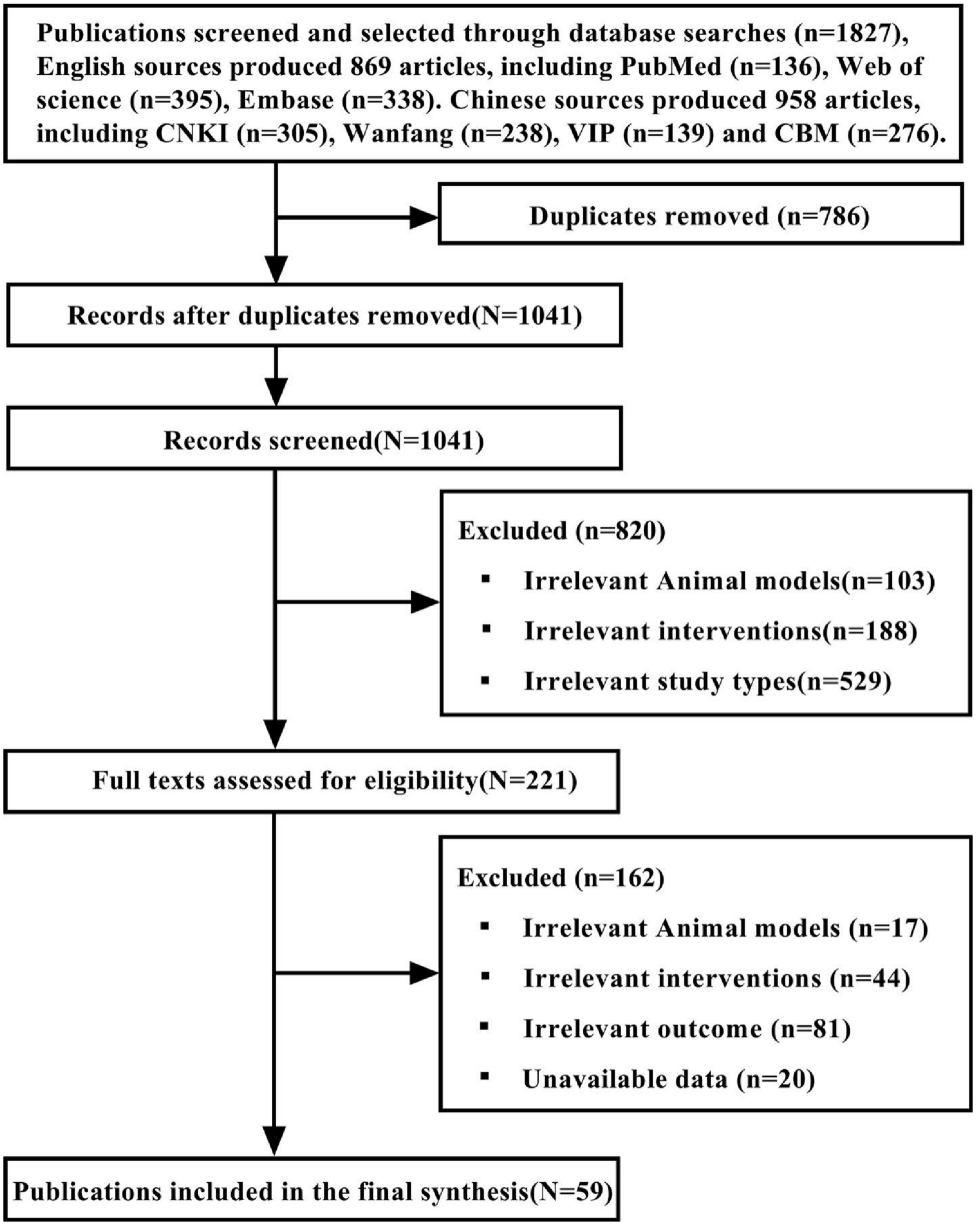
Flowchart of references - screening process.

The search of the relevant literature yielded a total of 1827 articles, with 958 of them being written in Chinese and 869 in English. Following the removal of duplicates and publications that did not fulfill the criteria for inclusion, 59 articles (29 English and 30 Chinese) assessing the efficacy of TCM monomers in SCI were finally included. Figure 1 provides an in-depth description of the screening process.

### 3.2. Basic study characteristics

**Figure 2:**
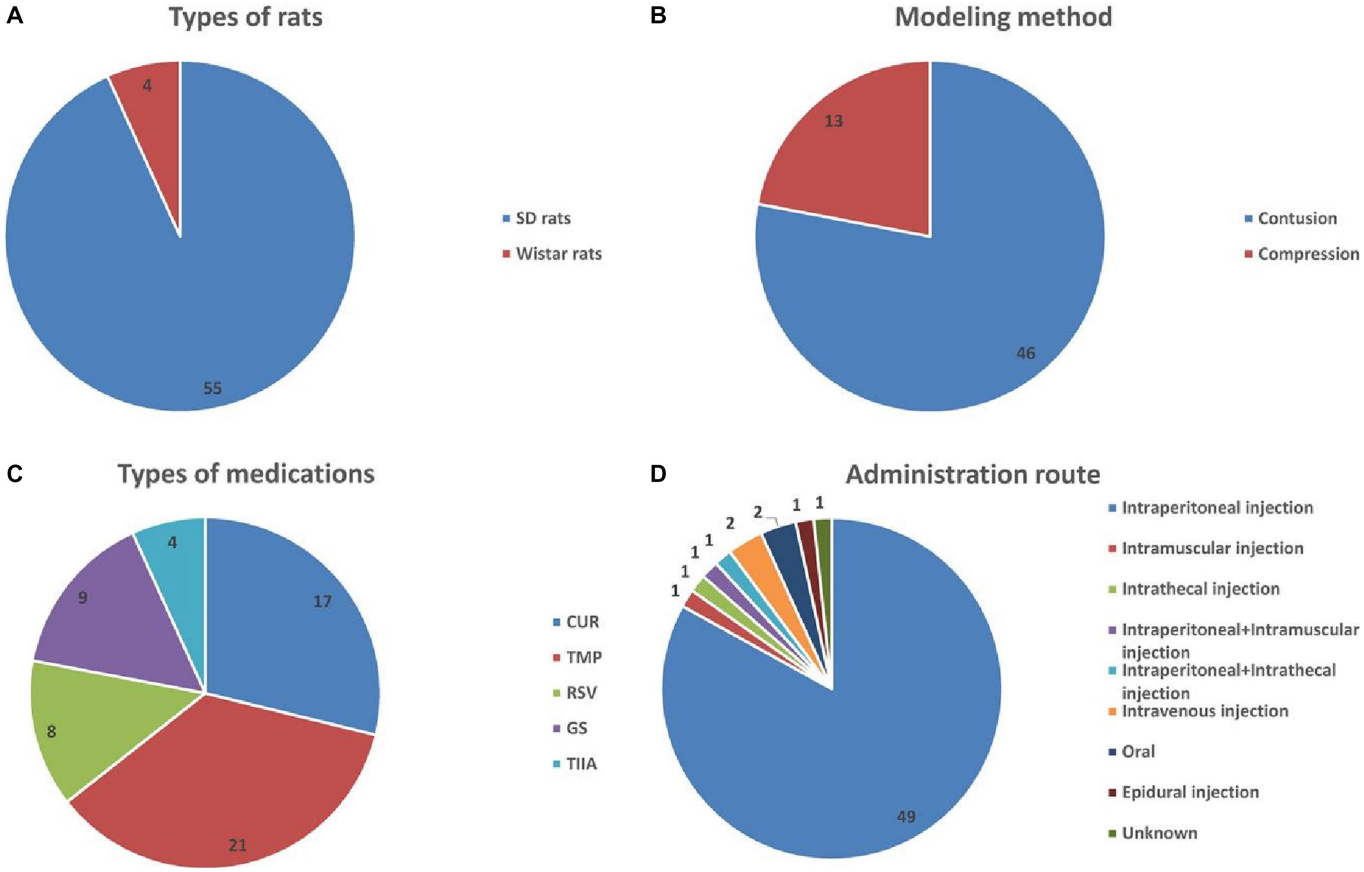
Basic information for inclusion in the study. **(A)** Types of rats; **(B)** modeling method; **(C)** types of medications; **(D)** administration route.

In total, 56 randomized controlled trials (RCTs) and three controlled studies were included. Animals used in these investigations were either SD rats (55 articles) or Wistar rats (4 articles), see Figure 2A. In total, 27 publications used only male rats, 21 used only female rats, and 8 comprised equal numbers of male and female rats; In three of the research studies, the rat’s sex was not specified. Overall, rats varied in age between 6 weeks and 36 weeks, in weight between 180 g and 350 g, and in sample size between 4 and 54. Contusion (46 research articles) and compression (13 articles) were the two modes of modeling used, see Figure 2B. Regarding the TCM monomers, 17, 21, 8, 9, and 4 studies evaluated the efficacy of CUR, TMP, RSV, GS, and TIIA, respectively, see Figure 2C. The monomers were administered intraperitoneally (49 studies), intramuscularly (1 study), intrathecally (1 study), intraperitoneally + intramuscularly (1 study), intraperitoneally + intrathecally (1 study), intravenously (2 studies), orally (2 studies), or epidurally (1 study); the route of administration was not reported in 1 study, see Figure 2D. Supplementary Table S2 displays the included publications’ baseline characteristics.

### 3.3. Risk of bias

**Figure 3:**
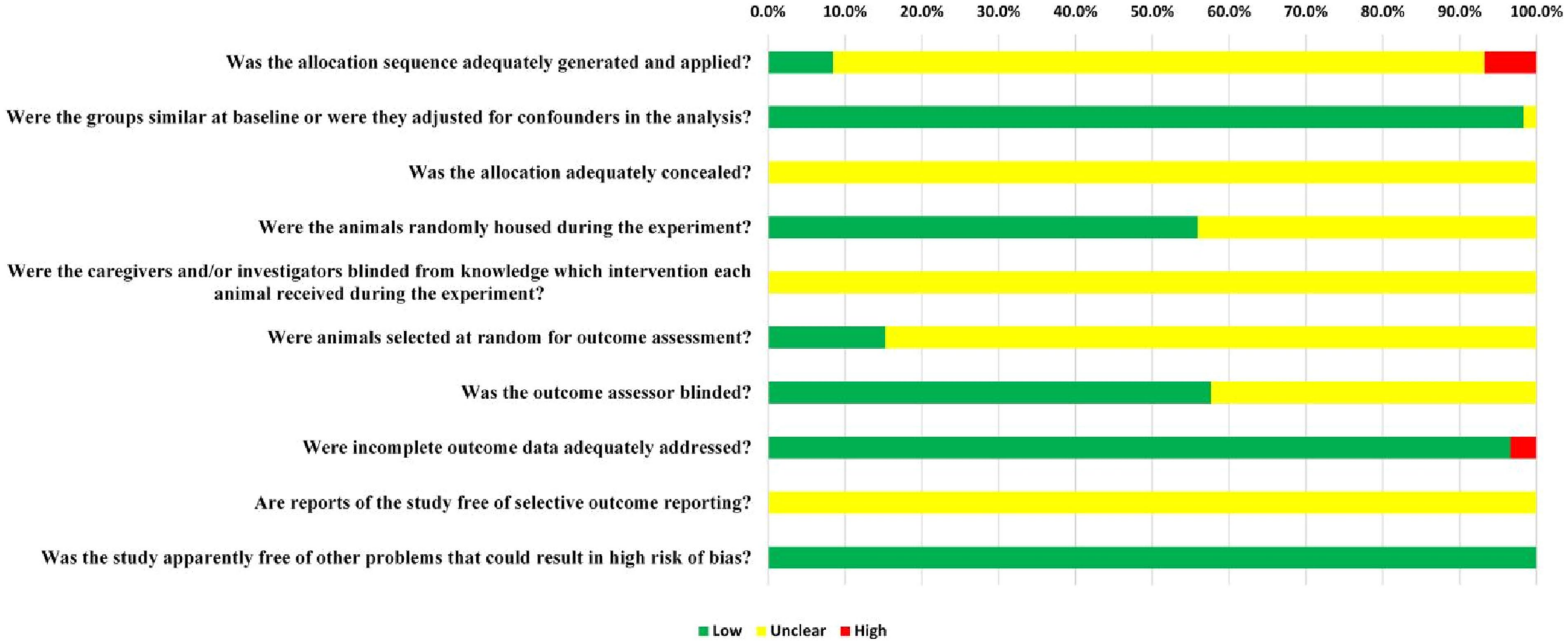
The results of the risk of bias assessment.

The assessment results showed a medium quality for all the included literatures. Only five of the 56 RCTs (8.5%) that were examined provided evidence that randomization was carried out with the aid of a random number table or a computer. However, in these publications, the use of concealed grouping was not reported at any point. Overall, 98.3% of the articles (58 out of 59) indicated that the subjects’ baseline characteristics, including age, sex, and body weight, were matching. Moreover, the random allocation of rats throughout the experiment was indicated by 55.9% (33/59) of the reports. Since the included publications only provided a limited amount of information, it was not possible to obtain blinding information from those publications. However, only 15.3% of studies (9/59) indicated they randomly selected animals for measuring outcomes. Blinding of outcome assessors was applied in 57.6% (34/59) of studies. In 96.6% (57/59) of the studies, all rats were included in the final analysis. The purity of TCM monomers has not been reported in any studies. All expected results were clearly reported, but not all studies provided access to the protocol. Therefore, there is a high risk of performance bias in literature quality assessment. Figure 3 provides a comprehensive summary of the methods used to evaluate the potential for bias across studies.

### 3.4. Network meta-analysis

#### 3.4.1. First week after treatment with TCM monomers

**Figure 4:**
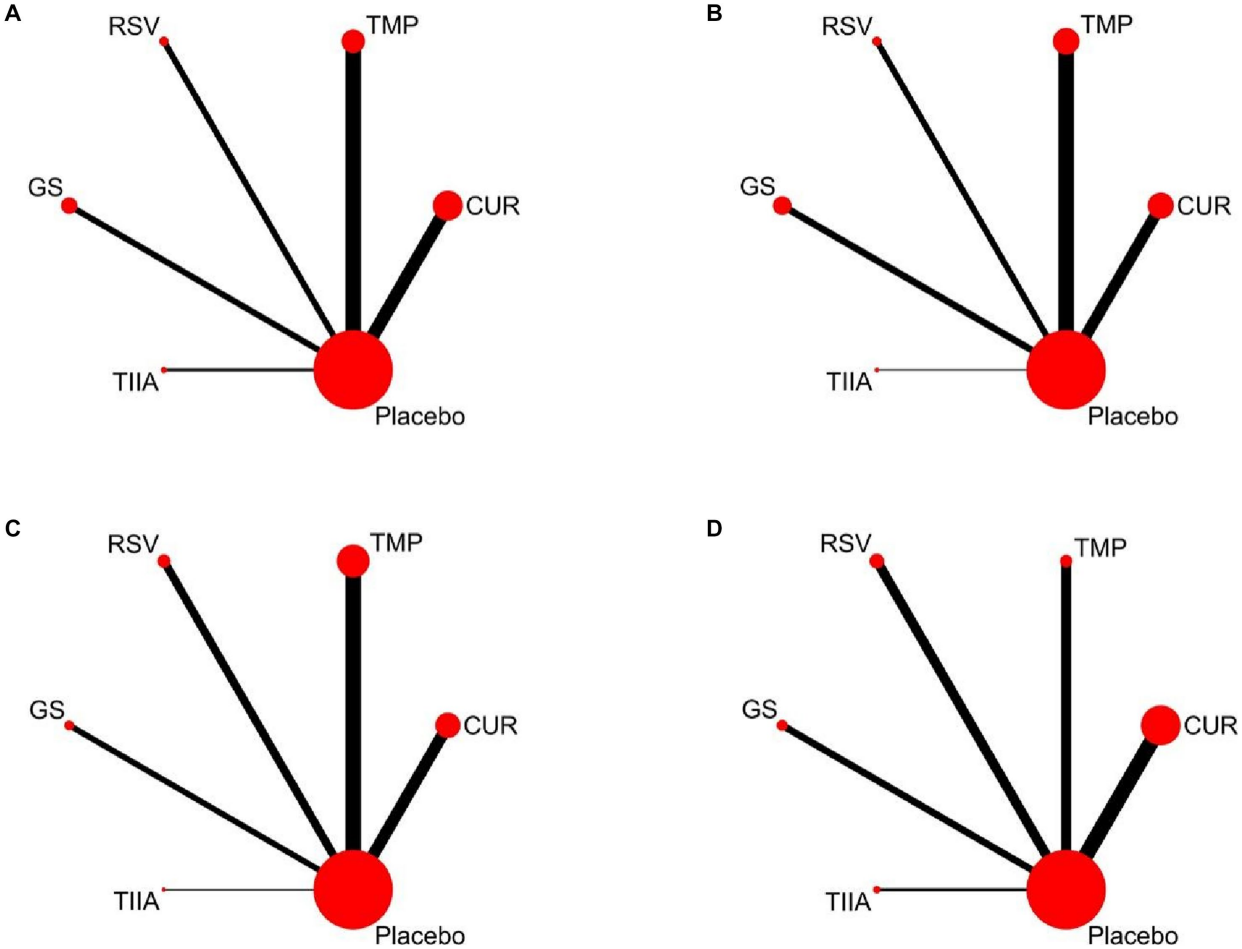
Evidence network diagram Circle size represents sample size involved; thickness of the line segment represents the number of studies involving both interventions. **(A)** The first week; **(B)** the second week; **(C)** the third week; **(D)** the fourth week.

**Figure 5:**
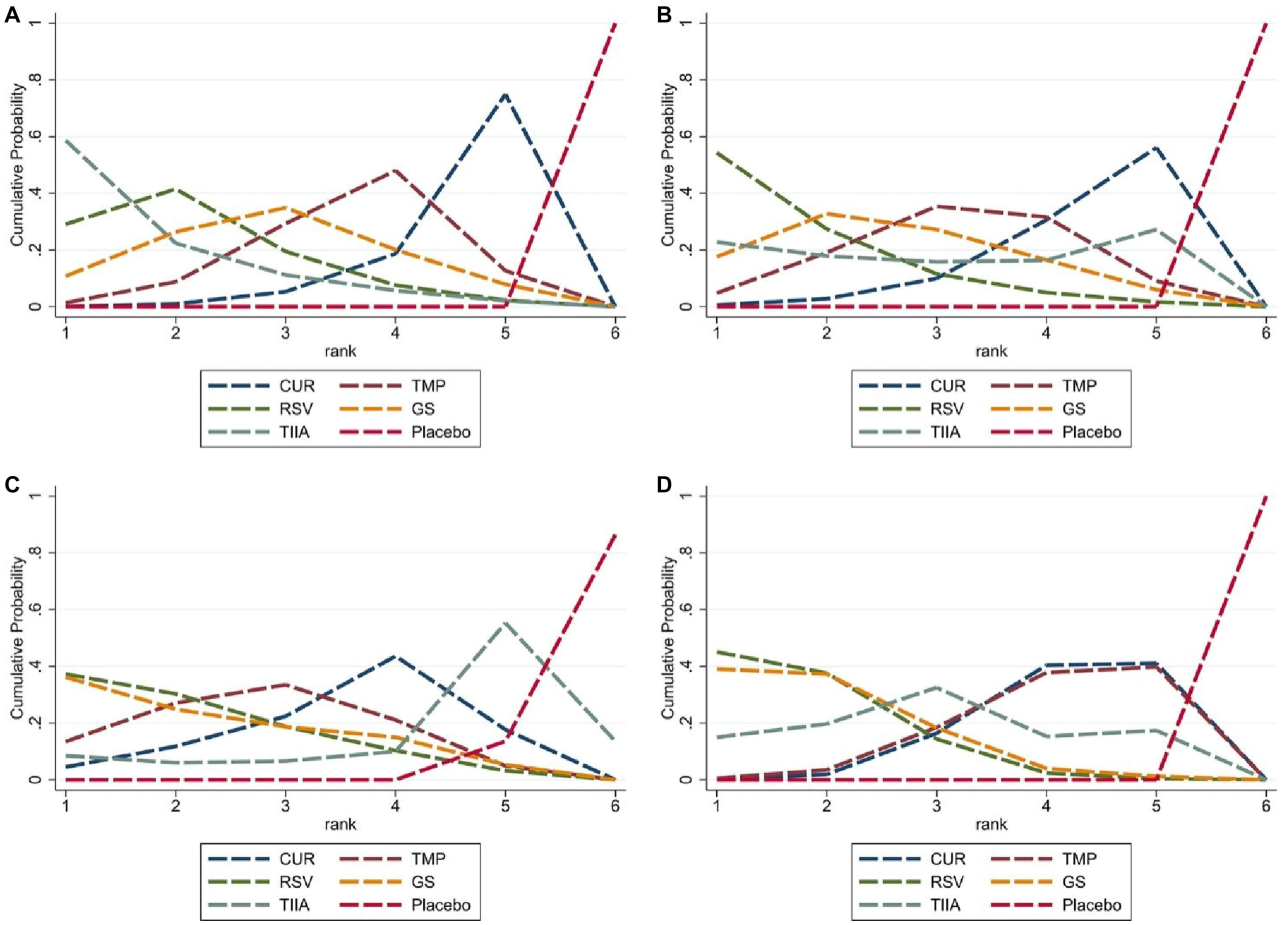
Cumulative probability ranking curve. The vertical axis represents cumulative probabilities, while the horizontal axis represents ranks. **(A)** The first week; **(B)** the second week; **(C)** the third week; **(D)** the fourth week.

**Figure 6:**
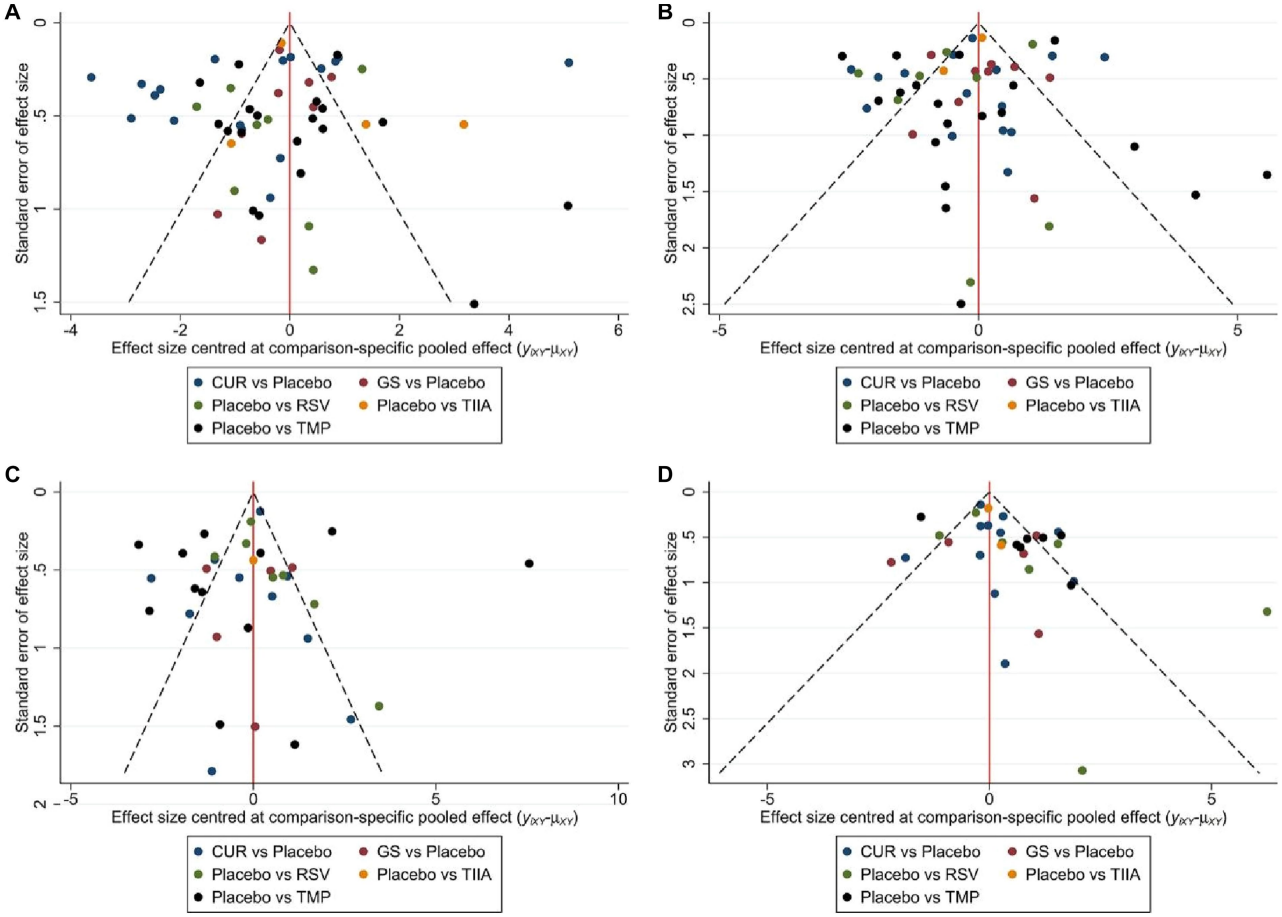
The comparison-correction funnel plot **(A)** The first week. **(B)** The second week. **(C)** The third week. **(D)** The fourth week.

A total of 57 studies were included for network meta-analysis. The evidence network showed there was no direct comparison between all types of TCM monomers. At the same time, the number of studies on TMP was the largest, see Figure 4A. The results of NMA indicated that rats had significantly higher BBB scores in TCM monomer groups compared to negative controls. However, the differences in BBB scores of rats between the five types of TCM monomers were not statistically significant, see Table 1. Rank ordering and SUCRA value results showed that TIIA might be the most effective TCM monomer for SCI, see Figure 5A and Supplementary Table S3. The comparison-correction funnel plot was basically symmetrical, suggesting that there was less possibility of publication bias, see Figure 6A.

**Table 1:** Network meta-analysis results 1 week after treatment with TCM monomers.

CUR					
−0.53 (−1.58,0.52)	TMP				
−1.28 (−2.67,0.10)	−0.75 (−2.13,0.62)	RSV			
−0.89 (−2.25,0.47)	−0.36 (−1.71,0.98)	0.39 (−1.23,2.02)	GS		
−1.67 (−3.41,0.07)	−1.14 (−2.87,0.59)	−0.38 (−2.34,1.57)	−0.78 (−2.71,1.16)	TIIA	
**1.75 (1.00,2.50)**	**2.28 (1.55,3.01)**	**3.04 (1.87,4.20)**	**2.64 (1.51,3.77)**	**3.42 (1.85,4.99)**	Placebo

Bold values are significant pairwise comparisons.

#### 3.4.2. Second week after treatment with TCM monomers

A total of 54 studies were included for network meta-analysis. The evidence network showed there was no direct comparison between all types of TCM monomers. At the same time, the number of studies on TMP was the largest, see Figure 4B. The results of NMA indicated that rats had significantly higher BBB scores in TCM monomer groups compared to negative controls. However, the differences in BBB scores of rats between the five types of TCM monomers were not statistically significant, see Table 2. Rank ordering and SUCRA value results showed that RSV might be the most effective TCM monomer for SCI, see Figure 5B and Supplementary Table S4. The comparison-correction funnel plot was basically symmetrical, suggesting that there was less possibility of publication bias, see Figure 6B.

**Table 2:** Network meta-analysis results 2 week after treatment with TCM monomers.

CUR					
−0.49 (−1.47,0.49)	TMP				
−1.15 (−2.39,0.10)	−0.66 (−1.88,0.56)	RSV			
−0.73 (−1.89,0.43)	−0.24 (−1.38,0.90)	0.42 (−0.96,1.79)	GS		
−0.53 (−2.47,1.41)	−0.04 (−1.97,1.88)	0.61 (−1.45,2.68)	0.20 (−1.82,2.22)	TIIA	
**2.89 (2.17,3.60)**	**3.37 (2.70,4.05)**	**4.03 (3.01,5.05)**	**3.62 (2.70,4.54)**	**3.42 (1.62,5.22)**	Placebo

Bold values are significant pairwise comparisons.

#### 3.4.3. Third week after treatment with TCM monomers

A total of 36 studies were included for network meta-analysis. The evidence network showed there was no direct comparison between all types of TCM monomers. At the same time, the number of studies on TMP was the largest, see Figure 4C. The results of NMA indicated that rats had significantly higher BBB scores in TCM monomer groups compared to negative controls. However, the differences in BBB scores of rats between the five types of TCM monomers were not statistically significant, see Table 3. Rank ordering and SUCRA value results showed that RSV might be the most effective TCM monomer for SCI, see Figure 5C and Supplementary Table S5. The comparison-correction funnel plot was basically symmetrical, suggesting that there was less possibility of publication bias, see Figure 6C.

**Table 3:** Network meta-analysis results 3 week after treatment with TCM monomers.

CUR					
−0.53 (−2.33,1.26)	TMP				
−0.97 (−3.04,1.10)	−0.44 (−2.40,1.52)	RSV			
−0.91 (−3.25,1.42)	−0.38 (−2.62,1.86)	0.06 (−2.40,2.53)	GS		
1.38 (−2.88,5.65)	1.92 (−2.29,6.13)	2.36 (−1.98,6.69)	2.29 (−2.17,6.76)	TIIA	
**3.69 (2.34,5.04)**	**4.23 (3.05,5.41)**	**4.67 (3.10,6.23)**	**4.60 (2.70,6.51)**	2.31 (−1.73,6.35)	Placebo

Bold values are significant pairwise comparisons.

#### 3.4.4. Fourth week after treatment with TCM monomers

A total of 32 studies were included for network meta-analysis. The evidence network showed there was no direct comparison between all types of TCM monomers. At the same time, the number of studies on CUR was the largest, see Figure 4D. The results of NMA indicated that rats had significantly higher BBB scores in TCM monomer groups compared to negative controls. Except for the CUR and RSV, the differences in BBB scores of rats between other types of TCM monomers were not statistically significant, see Table 4. Rank ordering and SUCRA value results showed that RSV might be the most effective TCM monomer for SCI, see Figure 5D and Supplementary Table S6. The comparison-correction funnel plot was basically symmetrical, suggesting that there was less possibility of publication bias, see Figure 6D.

**Table 4:** Network meta-analysis results 4 week after treatment with TCM monomers.

CUR					
−0.03 (−1.23,1.17)	TMP				
**−1.33 (−2.62,-0.04)**	−1.30 (−2.68,0.08)	RSV			
−1.26 (−2.66,0.14)	−1.23 (−2.73,0.27)	0.07 (−1.50,1.64)	GS		
−0.63 (−2.43,1.17)	−0.60 (−2.48,1.27)	0.70 (−1.24,2.63)	0.63 (−1.39,2.65)	TIIA	
**3.17 (2.41,3.92)**	**3.20 (2.27,4.12)**	**4.50 (3.46,5.54)**	**4.43 (3.25,5.61)**	**3.80 (2.16,5.43)**	Placebo

Bold values are significant pairwise comparisons.

## 4. Discussion

As far as we know, this is the first NMA that evaluates the motor function recovery in rat blunt SCI models following treatment with TCM monomers The BBB Locomotor Rating scale (score range: 0–21; complete flaccid paraplegia to normal function) is a sensitive measure of motor function recovery. Accordingly, this tool was used for assessing motor function recovery in SCI rat models (30–32). According to NMA, almost all TCM monomers significantly improved motor function recovery when compared with the negative control group at weeks 1, 2, 3, and 4. This finding indicates the considerable potential of TCM monomers in treating SCI. Although the lack of statistical significance in the BBB scores of the five TCM monomer groups at weeks 1, 2, and 3 may be attributed to the small sample size, the results based on rank ordering and SUCRA values still indicate that in the first week after SCI, the TIIA group showed the best recovery of motor function, while the RSV group exhibited the best recovery of motor function in the last 3 weeks. Therefore, we can consider TIIA may be the most effective TCM monomer during the first week, whereas RSV may be the most effective TCM monomer during the last 3 weeks in promoting motor function recovery. In the future, further studies with larger sample sizes are needed to validate our findings.

Several key conclusions emerge from the studies evaluated in this meta-analysis. TIIA may have a better effect on early recovery of motor function than the other monomers. This conclusion has been supported by a few various researchers. Our team established in a previous research report that TIIA has great potential in remodeling the spinal pathway and exhibits neuroprotective effects in the early stage of SCI (33). In addition, TIIA can relieve histopathological damage, rescue microvessels, and reduce blood–brain barrier permeability to protect motor neurons through the Notch signaling pathway (34). In SCI, an increase in the number of activated astrocytes and glial scarring both lead to decreased neurological function. Treatment with TIIA can thus effectively ameliorate these biological changes leading to a satisfactory functional recovery (35). Therefore, TIIA exhibits neuroprotective activity and promotes recovery of motor function in the early stage of SCI. However, caution should be exercised in assessing whether the early therapeutic effect of TIIA in SCI is the most optimal among the five TCM monomers, as this study only included four eligible studies on TIIA treatment for SCI. Further relevant literature is still needed to validate this conclusion.

In addition, according to the findings of this investigation, RSV could be the TCM monomer that is most efficient in promoting the restoration of motor function in rats with blunt SCI during the last 3 weeks of treatment. Secondary injury, including inflammation, oxidative stress responses, and neuronal apoptosis, occurring after the initial SCI results in further neurological damage. This pathophysiological status is alleviated by RSV owing to its ability to relieve inflammation, inhibit oxidative damage, and prevent apoptosis. Furthermore, previous studies have demonstrated that RSV suppresses the inflammatory response in SCI by upregulating the SIRT-1 signaling pathway and downregulating the NF-κB signaling pathways (36, 37). SCI is associated with the generation of free radicals, which cause oxidative degradation of lipids (38). Therefore, malondialdehyde (MDA) and superoxide dismutase (SOD) are often used as indices of oxidative injury in SCI (39). Prior studies have found that RSV is a good biological antioxidant that can reduce secondary oxidative stress-induced cell injury after SCI. It mediates this activity by increasing the SOD level and decreasing the MDA level in serum (40). A few studies have found that treatment with RSV is associated with significant upregulation of the anti-apoptotic gene Bcl-2 as well as significant inhibition of neuronal apoptosis (41, 42). These findings have boosted the potential of RSV as a treatment for SCI. The results of this study further support these findings that RSV is a promising drug for promoting motor function recovery after blunt SCI.

The other three TCM monomers (CUR, TMP, and GSs) can also play an indispensable function in SCI treatment. Studies have shown that CUR inhibits the overactivation of microglia by regulating the expression of microglia TLR4, thereby reducing inflammation-induced neuronal injury (43). In addition, CUR activates the Nrf2 signaling pathway and upregulates the Nrf2/HO-1 signaling pathway, which promotes free radical antioxidant properties (44). Thus, CUR has demonstrated efficacy in alleviating inflammation and oxidative damage associated with central nervous system damage in mammals (45). Similarly, *in vivo* data have shown that TMP regulates the spinal cord microenvironment (46). In a rat model of acute SCI, TMP decreases the expression of migration inhibitory factors, which may play a role in repairing damaged tissue (47). A few studies have suggested that TMP inhibits the expression of IL-18, IL-1β, TNF-α, NF-κB, and neutrophil infiltration and increases the level of NF-κB inhibitor and IL-10. These activities reduce the inflammatory response after SCI and exert a neuroprotective activity (48, 49). Similarly, the efficacy of GSs in treating SCI has been demonstrated. By inducing neurotrophic factors for astroglia, GSs have demonstrated enhancement of scratch wound healing in cell cultures; moreover, GSs have shown improvement in nerve function recovery in animal models of SCI (45, 50). In addition, GS can effectively inhibit the SCI-induced activation of the MAPK signaling pathway, thus alleviating secondary injury after SCI (51). These findings can well explain the mechanism of the three TCM monomers promoting motor function recovery following blunt SCI, which can lay a foundation for future research.

TCM monomers thus exhibit neuroprotective activity and could remarkably enhance motor function in rats with blunt SCI, especially TIIA, and RSV. Furthermore, combining multiple therapeutic approaches would benefit spinal cord functional recovery; these include the use of two or more TCM monomers and the use of TCM monomers combined with molecular therapy, cell therapy, or tissue engineering. Therefore, in the future, TCM monomers will be an important focus in the quest for blunt SCI treatment.

## 5. Limitations

This research faced several shortcomings. Firstly, the data that are now available on TCM monomers and the decline in motor function linked to SCI are inadequate because of the restricted number of rats involved in each trial, the limited sample sizes, and the insufficient examination of the data on BBB scores. Secondly, We only considered the BBB score since it is the most common and best shows the impact of TCM monomers treatment; however, we failed to assess any further outcome measures due to few reports. Thirdly, we were unable to determine where the heterogeneity originated in an accurate manner. Therefore, in our investigation, we used a model with random effects, which resulted in highly conservative findings. Fourth, this study compared five TCM monomers that are commonly used for the treatment of SCI; thus, the potential therapeutic role of other TCM monomers may have been overlooked. Fifth, drug dose and administration route may have affected the recovery of motor function. However, subgroup analyses were not performed owing to a smaller number of included studies. Sixth, the purity of TCM monomer is closely related to the therapeutic effect and dosage, but it has not been reported in any study, which may bring a certain risk of bias; In addition, the number of animals in different studies varies, and the quality of the literature included in the studies needs to be carefully evaluated.

## 6. Conclusion

The NMA revealed that TCM monomers could effectively restore motor function in the rat model of blunt SCI. TIIA may be the most effective TCM monomer to improve motor function recovery in the first week of rats with blunt SCI, and RSV may be the most effective TCM monomer during the last 3 weeks.

The existing animal experiments on the use of TCM monomers for SCI still encounter various difficulties with blinding, allocation concealment, randomization, and reporting of results, according to a systematic review of the included research. Because of these limitations, animal research may not provide reliable findings. The quality of evidence in preclinical investigations may be improved by standardizing the implementation and reporting of animal experiments, and the risk of extrapolating preclinical findings into clinical settings can be reduced.

## Data availability statement

The original contributions presented in the study are included in the article/Supplementary material, further inquiries can be directed to the corresponding author.

## Author contributions

LX conceived the study. LX, YY, WZ, WL, CL, ZG, and XY contributed to the study design. LX drafted the manuscript. YY and XY edited the manuscript. All authors contributed to the article and approved the submitted version.

## Funding

This work was funded by the National Natural Science Foundation of the People’s Republic of China (No. 81973882).

## Conflict of interest

The authors declare that the research was conducted in the absence of any commercial or financial relationships that could be construed as a potential conflict of interest.

## Publisher’s note

All claims expressed in this article are solely those of the authors and do not necessarily represent those of their affiliated organizations, or those of the publisher, the editors and the reviewers. Any product that may be evaluated in this article, or claim that may be made by its manufacturer, is not guaranteed or endorsed by the publisher.

## Supplementary material

The Supplementary material for this article can be found online at: https://www.frontiersin.org/articles/10.3389/fneur.2023.1165076/full#supplementary-material
