# Supplementary material for: Comparative efficacy of five most common traditional Chinese medicine monomers for promoting recovery of motor function in rats with blunt spinal cord injury: a network meta-analysis
Source: Front Neurol. 2023 Jul 3;14:1165076. doi: 10.3389/fneur.2023.1165076 (PMC10351986; doi:10.3389/fneur.2023.1165076)
Supplement: Supplementary file 1 [file Table_1.DOCX]

**Comparative Efficacy of Five Most Common Traditional Chinese Medicine Monomers for promoting recovery of motor function in Rats with Blunt Spinal Cord Injury: A Network Meta-Analysis**

**Table S1: Chinese and English search strategies**

| **PubMed**  #1"Spinal Cord Injuries"[Mesh] 54518  #2"Spinal cord injury"[Title/Abstract] OR "Spinal injury"[Title/Abstract] OR "Spinal Cord Trauma"[Title/Abstract] OR "Cord Trauma, Spinal"[Title/Abstract] OR "Cord Traumas, Spinal"[Title/Abstract] OR "Spinal Cord Traumas"[Title/Abstract] OR "Trauma, Spinal Cord"[Title/Abstract] OR "Traumas, Spinal Cord"[Title/Abstract] OR "Myelopathy, Traumatic"[Title/Abstract] OR "Myelopathies, Traumatic"[Title/Abstract] OR "Traumatic Myelopathies"[Title/Abstract] OR "Traumatic Myelopathy"[Title/Abstract] OR "Injuries, Spinal Cord"[Title/Abstract] OR "Cord Injuries, Spinal"[Title/Abstract] OR "Cord Injury, Spinal"[Title/Abstract] OR "Injury, Spinal Cord"[Title/Abstract] OR "Spinal Cord Transection"[Title/Abstract] OR "Spinal Cord Laceration"[Title/Abstract] OR "Cord Laceration, Spinal"[Title/Abstract] OR "Cord Lacerations, Spinal"[Title/Abstract] OR "Laceration, Spinal Cord"[Title/Abstract] OR "Lacerations, Spinal Cord"[Title/Abstract] OR "Post-Traumatic Myelopathy"[Title/Abstract] OR "Myelopathies, Post-Traumatic"[Title/Abstract] OR "Myelopathy, Post-Traumatic"[Title/Abstract] OR "Post Traumatic Myelopathy"[Title/Abstract] OR "Post-Traumatic Myelopathies"[Title/Abstract] OR "Contusion, Spinal Cord"[Title/Abstract] OR "Contusions, Spinal Cord"[Title/Abstract] OR "Cord Contusion, Spinal"[Title/Abstract] OR "Cord Contusions, Spinal"[Title/Abstract] OR "Spinal Cord Contusions"[Title/Abstract] OR "Spinal Cord Contusion"[Title/Abstract] 45389  #3 #1 OR #2 69230  #4 "Curcumin"[Mesh] 13900  #5 "turmeric yellow"[Title/Abstract] OR "yellow turmeric"[Title/Abstract] OR "curcumin phytosome"[Title/Abstract] OR "Diferuloylmethane"[Title/Abstract] 398  #6 #4 OR #5 13963  #7 "Ginsenosides"[Mesh] 4814  #8 "Panaxosides"[Title/Abstract] OR "Ginsenoside"[Title/Abstract] 5168  #9 #7 OR #8 6494  #10 "Resveratrol"[Mesh] 10433  #11 "3 5 4 trihydroxystilbene"[Title/Abstract] OR "3 4 5 trihydroxystilbene"[Title/Abstract] OR "trans-Resveratrol-3-O-sulfate"[Title/Abstract] OR "SRT-501"[Title/Abstract] OR "cis-Resveratrol"[Title/Abstract] OR "trans-Resveratrol"[Title/Abstract] OR "Resveratrol-3-sulfate"[Title/Abstract] 1626  #12 #10 OR #11 10902  #13 "tanshinone" [Supplementary Concept] 1391  #14 "tanshinone i"[Title/Abstract] OR "TTE-50"[Title/Abstract] OR "tanshinone ii a"[Title/Abstract] OR "tanshinone iia"[Title/Abstract] OR "tanshinone ii b"[Title/Abstract] OR "tanshinone iib"[Title/Abstract] 1737  #15 #13 OR #14 2055  #16 "tetramethylpyrazine" [Supplementary Concept] 1029  #17 "ligustrazine"[Title/Abstract] OR "TMPZ"[Title/Abstract] OR "chuanxiongzine"[Title/Abstract] OR "tetramethyl pyrazine"[Title/Abstract] OR "tetramethylpyrazine hydrochloride"[Title/Abstract] OR "Liqustrazine"[Title/Abstract] 598  #18 #16 OR #17 1183  #19 #6 OR #9 OR #12 OR #15 OR #18 34123  #20 #3 AND #19 136  **Web of science**  TS=(Spinal cord injury OR Spinal Cord Injuries OR Spinal injury OR Spinal Cord Trauma OR Spinal Cord Transection OR Spinal Cord Laceration OR Post-Traumatic Myelopathy OR Spinal Cord Contusion) AND TS=(curcumin OR Turmeric Yellow OR Yellow, Turmeric OR Curcumin Phytosome OR Diferuloylmethane OR ginsenosides OR Panaxosides OR Ginsenoside OR Resveratrol OR 3,5,4'-Trihydroxystilbene OR 3,4',5-Trihydroxystilbene OR trans-Resveratrol-3-O-sulfate OR SRT-501 OR cis-Resveratrol OR trans-Resveratrol OR Resveratrol-3-sulfate OR tanshinone OR tanshinone I OR TTE-50 OR tanshinone II A OR tanshinone IIA OR tanshinone II B OR tanshinone IIB OR Tetramethylpyrazine OR ligustrazine OR TMPZ OR chuanxiongzine OR tetramethylpyrazine OR tetramethylpyrazine hydrochloride OR Liqustrazine) 395  **Embase**  #1: spinal cord injury'/exp 90,788  #2. 'spinal injury':ab,ti 5,711  #3. 'spinal cord injuries':ab,ti 7,192  #4. 'spinal cord injury':ab,ti 52,646  #5. 'spinal cord trauma':ab,ti 1,109  #6. 'spinal cord transection':ab,ti 1,763  #7. 'spinal cord laceration':ab,ti 4  #8. 'post-traumatic myelopathy':ab,ti 16  #9. 'spinal cord contusion':ab,ti 917  #10. #1 OR #2 OR #3 OR #4 OR #5 OR #6 OR #7 OR #8 OR #9 100,202  #11. 'curcumin'/exp 33,211  #12. 'turmeric yellow':ab,ti 4  #13. 'yellow, turmeric':ab,ti 1  #14. 'curcumin phytosome':ab,ti 12  #15. 'diferuloylmethane':ab,ti 498  #16. #11 OR #12 OR #13 OR #14 OR #15 33,224  #17. 'ginsenosides'/exp 5,090  #18. 'panaxosides':ab,ti 5  #19. 'ginsenoside':ab,ti 6,126  #20. #17 OR #18 OR #19 8,611  #21. 'resveratrol'/exp 27,223  #22. 'trans-resveratrol-3-o-sulfate':ab,ti 15  #23. 'srt-501':ab,ti 2  #24. 'cis-resveratrol':ab,ti 139  #25. 'trans-resveratrol':ab,ti 1,342  #26. 'resveratrol-3-sulfate':ab,ti 19  #27. #21 OR #22 OR #23 OR #24 OR #25 OR #26 27,365  #28. 'tanshinone'/exp 1,340  #29. 'tanshinone i':ab,ti 419  #30. 'tte-50':ab,ti 10  #31. 'tanshinone ii a':ab,ti 432  #32. 'tanshinone iia':ab,ti 1,695  #33. 'tanshinone ii b':ab,ti 3  #34. 'tanshinone iib':ab,ti 26  #35. #28 OR #29 OR #30 OR #31 OR #32 OR #33 OR #34 2,989  #36. 'tetramethylpyrazine'/exp 2,050  #37. 'ligustrazine':ab,ti 743  #38. 'tmpz':ab,ti 33  #39. 'chuanxiongzine':ab,ti 13  #40. 'tetramethyl pyrazine':ab,ti 32  #41. 'tetramethylpyrazine hydrochloride':ab,ti 18  #42. 'liqustrazine':ab,ti 3  #43. #36 OR #37 OR #38 OR #39 OR #40 OR #41 OR #42 2,125  #44. #16 OR #20 OR #27 OR #35 OR #43 69,409  #45. #10 AND #44 338  **CNKI/高级检索; CNKI / Advanced Search**  主题:脊髓损伤 AND (姜黄素 OR 人参皂苷 OR 白藜芦醇 OR 丹参酮 OR 川芎嗪) (305)  Subject: spinal cord injury AND (curcumin OR ginsenoside OR Resveratrol OR tanshinone OR Tetramethylpyrazine) (305)  **万方/高级检索; Wanfang database/ Advanced Search**  主题:脊髓损伤 AND (姜黄素 OR 人参皂苷 OR 白藜芦醇 OR 丹参酮 OR 川芎嗪) (238)  Subject: spinal cord injury AND (curcumin OR ginsenoside OR Resveratrol OR tanshinone OR Tetramethylpyrazine) (238)  **VIP/高级检索; VIP database/ Advanced Search**  题名或关键词: 脊髓损伤 AND (姜黄素 OR 人参皂苷 OR 白藜芦醇 OR 丹参酮 OR 川芎嗪) (139)  Title or keyword: spinal cord injury AND (curcumin OR ginsenoside OR Resveratrol OR tanshinone OR Tetramethylpyrazine) (139)  **CBM/高级检索; CBM / Advanced Search**  #1:" Spinal cord injury "[unweighted, extended] 62455  #2:" Spinal cord injury "[common field: smart] 81316  #3:#1 OR #2 81316  #4:"curcumin"[unweighted, extended] 25560  #5: "curcumin"[common field: smart] 18341  #6:"ginsenosides"[unweighted, extended] 4562  #7: "ginsenoside"[common field: smart] 14525  #8:"Resveratrol"[unweighted, extended] 3230  #9: "Resveratrol"[common field: smart] 19950  #10:"tanshinone"[unweighted, extended] 5331  #11: "tanshinone"[common field: smart] 9160  #12:"Tetramethylpyrazine"[unweighted, extended] 7421  #13: "Tetramethylpyrazine"[common field: smart] 10463  #14:#4 OR #5 OR #6 OR #7 OR #8 OR #9 OR #10 OR #11 OR #12 OR #13 78090  #15:#3 AND #14 276 |
| --- |

**Table S2: Basic information of included studies**

| **Number** | **Author,**  **year** | **Country** | **Type of study** | **Baseline characteristics** | | | | **Sample size（Experience/Control）** | **Model** | | | **Interventions** | | | **Follow up** |
| --- | --- | --- | --- | --- | --- | --- | --- | --- | --- | --- | --- | --- | --- | --- | --- |
|  |  |  |  | **Species** | **Gender** | **Weight** | **Age** |  | **Injury pathway** | **Location** | **Damage energy** | **Medication** | **Concentration/dose** | **Route** |  |
| 1 | Fan,  2023 | China | RCT | SD rats | / | 200±20g | / | 12/12 | Contusion | T9-11 | / | TMP | 80mg/kg,once per day for 28d | IP | 1w,2w,3w,4w |
| 2 | Su,  2022 | China | RCT | SD rats | Female | 200-220g | 8w | 13/13 | Contusion | T10 | 60gcm | CUR | 100mg/kg,once per day for 7d | IP | 1w,2w,4w |
| 3 | Shen,  2022 | China | RCT | SD rats | Female | 180-220g | Adult | 10/10 | Contusion | T9-10 | 40gcm(10g×4cm) | GS Rg1 | 100mg/kg,once per day,daily for consecutive days | IP | 1w,2w,3w,4w |
| 4 | Zhang,  2022 | China | RCT | SD rats | Female | 250±20g | 8w | 6/6 | Contusion | T10 | 50gcm(10g×5cm) | GS Rg1 | 10mg/kg,once per day for 14d | IP | 1w,2w,3w,4w |
| 5 | Li,  2021 | China | Control | SD rats | Male | 235±15g | 8w | 10/10 | Contusion | T9-11 | 20gcm(10g×2cm) | CUR | 60mg/kg,30min following the contusion and continued weekly for 3 weeks | IM | 1w,2w,3w,4w |
| 6 | Zhao,  2021 | China | RCT | SD rats | Female | 180-200g | 8-12w | 5/5 | Contusion | T9-10 | 30gcm(10g×3cm) | RSV | 30mg/kg,once per day for 7d | IP | 1w,2w,3w,4w |
| 7 | Chen,  2020 | China | RCT | SD rats | Female | 240-260g | / | 20/10 | Contusion | T9-10 | 50gcm(10g×5cm) | CUR | A.40mg/kg B.100mg/kg,once per day for 7d | IP | 1w |
| 8 | Zhang,  2020 | China | RCT | SD rats | Male | 200-230g | 6-8w | 6/6 | Contusion | T9 | 25gcm(10g×2.5cm) | TMP | 100mg/kg,once per day for 14d | IP | 1w,2w |
| 9 | Fan,  2020 | China | RCT | SD rats | Male | 230-250g | 16w | 5/5 | Contusion | / | / | RSV | 100mg/kg,once per day for 14d | IP | 1w,2w,4w |
| 10 | Liu,  2019 | China | RCT | SD rats | Male | 220-250g | Adult | 6/6 | Contusion | T9-10 | 25gcm(10g×2.5cm) | RSV | 100mg/kg,once per day for 35d | IP | 1w,2w,3w,4w |
| 11 | Yu,  2019 | China | RCT | SD rats | Female | 230g | Adult | 5/5 | Contusion | T9 | 50gcm(10g×5cm) | RSV | 50mg/kg after SCI,25mg/kg from the second day of SCI to the seventh day | IP | 1w,2w,3w,4w |
| 12 | Bang,  2018 | Korea | RCT | SD rats | Male | 290-310g | Adult | 20/20 | Compression | T9 | 30g,  2min | CUR | 10μL of 1μm per day for 7d | IT | 1w,2w,3w,4w |
| 13 | Ruzicka,  2018 | Czech Republic | RCT | Wistar rats | / | 300±15g | 12w | 12/19 | Compression | T8 | 15µL balloon,5min | CUR | 60mg/kg(IT), once a week,4 times;  6mg/kg(IP), once per day for 28d | IT+IP | 1w,2w,3w,4w |
| 14 | Ruzicka,  2018 | Czech Republic | RCT | Wistar rats | Male | 300±15g | 10w | 13/10 | Compression | T8 | 15µL balloon,5min | CUR | 60mg/kg(IM)(1,7, 14, 21 and 28 days post-SCI);  6 mg/kg(IP), once per day for 28d | IM+IP | 1w,2w,3w,4w |
| 15 | Meng,  2018 | China | Control | SD rats | Male | 250-300g | 8-12w | 5/5 | Contusion | T9-10 | 25gcm(10g×2.5cm) | RSV | 100mg/kg,single dose | IP | 1w,2w,3w |
| 16 | Wang,  2018 | China | RCT | SD rats | Female | 200-250g | Adult | 5/5 | Contusion | T10 | 25gcm(10g×2.5cm) | RSV | 200mg/kg,once per day for 3d | IP | 1w,2w,3w,4w |
| 17 | Liu,  2018 | China | RCT | SD rats | Female and Male | 220-260g | 7w | 10/10 | Contusion | T10 | 50gcm(10g×5cm) | GS Rb1 | 10mg/kg,once per day for 7d | IP | 1w,2w,3w,4w |
| 18 | Wang,  2018 | China | RCT | SD rats | Male | 200-220g | Adult | 6/6 | Compression | T7-10 | 30g,  1min | GS Rb1 | 20mg/kg,once per day for 28d | IP | 1w,2w,3w,4w |
| 19 | Li,  2018 | China | RCT | SD rats | Female | 200-220g | 8w | 5/5 | Contusion | T10 | 25gcm(10g×2.5cm) | GS Rg1 | 30mg/kg,once per day for 7d | IP | 1w,2w,3w,4w |
| 20 | Zhang,  2018 | China | RCT | SD rats | Female | 200-220g | 7w | 12/12 | Compression | T9-10 | 0.6N,  1min | TIIA | 30mg/kg,single dose before SCI;  20mg/kg,once per day after SCI for 7d | IV | 1w,2w,3w,4w |
| 21 | Hao,  2017 | China | RCT | SD rats | Female | 200-220g | Adult | 21/7 | Contusion | / | / | CUR | A.50mg/kg B.100mg/kg C.200mg/kg,single dose | IP | 1w,2w,3w,4w |
| 22 | Hao,  2017 | China | RCT | SD rats | Female | 200-220g | Adult | 7/7 | Contusion | T10 | 200 kdyn/cm^2^ | CUR | 100mg /kg,once per day for 3d | Oral | 1w,2w,3w,4w |
| 23 | Hu,  2017 | China | RCT | SD rats | Male | 180-220g | Adult | 5/5 | Contusion | T10 | 32gcm(8g×4cm) | TMP | 200mg/kg,once per day for 5d | IP | 1w,2w,3w,4w |
| 24 | Chen,  2017 | China | RCT | SD rats | / | / | / | 39/39 | Contusion | T10 | 28gcm(7g×4cm) | TMP | 200mg/kg,once per day for 5d | / | 1w,2w,3w |
| 25 | Zhao,  2017 | China | RCT | SD rats | Female | 220-240g | / | 5/5 | Contusion | T9-10 | 30gcm(10g×3cm) | RSV | 100mg/kg,single dose | IP | 1w,2w,3w,4w |
| 26 | Kim,  2017 | Korea | RCT | SD rats | Male | 280-300g | 12w | 36/18 | Compression | T10 | 35g,  5min | GS Rg3 | A.10mg/kg B.30mg/kg,once per day for 14d | Oral | 1w,2w |
| 27 | Yao,  2017 | China | RCT | SD rats | Male | 240-260g | 6-7w | 4/4 | Contusion | T9-11 | 25gcm(10g×2.5cm) | TIIA | 30mg/kg,once per day for 7d | IP | 1w |
| 28 | Yang,  2016 | China | RCT | SD rats | Female | 250-300g | Adult | 10/10 | Contusion | T9-11 | 9gcm(3g×3cm) | CUR | 200mg/kg,once per day for 7d | IP | 1w,2w,3w |
| 29 | Hu,  2016 | China | RCT | SD rats | Male | 250-300g | Adult | 5/5 | Contusion | T10 | 32gcm(8g×4cm) | TMP | 200mg/kg,once per day for 5d | IP | 1w,2w,3w,4w |
| 30 | Huang,  2016 | China | RCT | SD rats | Male | 180-220g | Adult | 4/4 | Contusion | T10 | 32gcm(8g×4cm) | TMP | 200mg/kg,once per day for 3d | IP | 1w,2w,3w,4w |
| 31 | Su,  2016 | China | RCT | SD rats | Female and Male | 300-350g | 12-24w | 10/10 | Compression | T2 | 30g,  1min | TMP | 200mg/kg,once per day for 14d | IP | 2w |
| 32 | Xiong,  2016 | China | RCT | SD rats | Male | 200-230g | Adult | 6/6 | Contusion | T9 | 50gcm(10g×5cm) | TMP | 200mg/kg,once per day for 10d | IP | 1w,2w,4w |
| 33 | Cong,  2016 | China | RCT | SD rats | Female | 250-300g | Adult | 54/18 | Contusion | T8 | 50gcm(10g×5cm) | GS Rd | A.12.5mg/kg B.25mg/kg C.50mg/kg,once per day for 14d | IP | 1w,2w |
| 34 | Yang,  2016 | China | RCT | SD rats | Female | 220-250g | Adult | 11/11 | Compression | T9 | 25gcm(10g×2.5cm) | TIIA | 20mg/kg,once per day for 7d | IV | 1w,2w,4w |
| 35 | Li,  2016 | China | RCT | SD rats | Female | 200g | Adult | 20/20 | Contusion | T9 | 50gcm(10g×5cm) | RSV | 100mg/kg,once per day | IP | 1w,2w,3w,4w |
| 36 | Machova,  2015 | Czech Republic | RCT | Wistar rats | Male | 300±15g | 10w | 5/5 | Compression | T9-10 | 15µL balloon,5min | CUR | 6 mg/kg,once per day for 28d | IP | 1w,2w,3w,4w |
| 37 | Hu,  2015 | China | RCT | SD rats | Male | 220-250g | Adult | 5/5 | Contusion | T10 | 24gcm(8g×3cm) | TMP | 80mg/kg,5 consecutive days from day 3 post-injury | IP | 1w,2w,3w,4w |
| 38 | Wang,  2015 | China | RCT | SD rats | Male | 250g | Adult | 10/10 | Contusion | T8 | 50gcm(10g×5cm) | TMP | 200mg/kg,once per day for 10d | IP | 1w,2w,3w |
| 39 | Zhang,  2015 | China | RCT | SD rats | Female and Male | 250±20g | Adult | 5/5 | Contusion | T9 | 50gcm(20g×2.5cm) | TMP | 200mg/kg,once per day for 7d | IP | 1w,2w |
| 40 | Sun,  2015 | China | RCT | SD rats | Male | 220-240g | 6-8w | 19/18 | Contusion | T8 | 25gcm | GS Rg1 | 10mg/kg,once per day for 14d | IP | 2w |
| 41 | Kim,  2014 | Korea | RCT | SD rats | Male | 290-310g | Adult | 8/8 | Compression | T9 | 30g,  2min | CUR | 200mg/kg,once per day for 7d | IP | 1w,2w |
| 42 | Yu,  2014 | China | RCT | SD rats | Male | 210-260g | / | 6/6 | Compression | T8-10 | 20g,  5min | CUR | 150mg/kg,once per day for 3d | IP | 1w,2w,3w |
| 43 | Zu,  2014 | China | RCT | SD rats | Male | 200-250g | Adult | 8/8 | Contusion | T8-10 | 150gcm(30g×5cm) | CUR | 40mg/kg,single dose | IP | 1w,2w |
| 44 | Xiang,  2014 | China | RCT | SD rats | Male | 250±20g | Adult | 30/30 | Compression | T9-10 | 70g,  1min | CUR | 200mg/kg,once per day for 7d | IP | 1w,2w,4w |
| 45 | Hu,  2013 | China | RCT | SD rats | Male | 250-300g | Adult | 5/5 | Contusion | T10 | 25gcm(5g×5 cm) | TMP | 200mg/kg,once per day for 5d | IP | 1w,2w,3w |
| 46 | Wang,  2013 | China | RCT | SD rats | Female and Male | 250-270g | Adult | 5/5 | Contusion | T10 | 25gcm(10g×2.5cm) | TMP | 200mg/kg,once per day for 5d | IP | 1w,2w,3w,4w |
| 47 | Qi,  2013 | China | RCT | SD rats | Female | 200-250g | / | 12/6 | Contusion | T9-10 | 50gcm(10g×5 cm) | CUR | A.40mg/kg B.100mg/kg,once per day for 7d | IP | 1w |
| 48 | Xiao,  2012 | China | RCT | SD rats | Female and Male | 250-300g | Adult | 5/5 | Contusion | T10 | 25gcm(5g×5cm) | TMP | 200mg/kg,once per day for 5d | IP | 1w,2w,3w |
| 49 | Yin,  2012 | China | RCT | SD rats | Male | 220-250g | Adult | 6/6 | Contusion | T9-11 | 25gcm(10g×2.5cm) | TIIA | 50 mg/kg,single dose before SCI; 20mg/kg,once per day after SCI for 7d | IP | 1w |
| 50 | Ormond,  2012 | USA | Control | SD rats | Female | 200-250g | Adult | 8/6 | Contusion | T9-10 | 12.5gcm(10g×1.25cm) | CUR | 60mg/kg,once per week for 6w | Epidural injection | 1w,2w,3w,4w |
| 51 | Zou,  2011 | China | RCT | Wistar rats | Female | 200±20g | 8w | 24/6 | Compression | T9-10 | 50g,  1min | CUR | A.300mg/kg,single dose;  B.30mg/kg C.100mg/kg  D.300mg/kg,once per day for 7d | IP | 1w,2w,4w |
| 52 | Qi,  2011 | China | RCT | SD rats | Male | 230±10g | Adult | 6/6 | Contusion | T9-11 | 40gcm(10g×4cm) | TMP | 40mg/per rat,single dose before SCI;  20mg/per rat,once per day after SCI for 14d | IP | 1w,2w |
| 53 | Zhou,  2010 | China | RCT | SD rats | Female and Male | 200-250g | Adult | 5/5 | Contusion | T10 | 20gcm(5g×4cm) | TMP | 200mg/kg,once per day for 5d | IP | 1w,2w,3w |
| 54 | Huang,  2010 | China | RCT | SD rats | Male | 250-300g | 24-36w | 10/10 | Contusion | T10 | 25gcm(5g×5cm) | TMP | 200mg/kg,once per day for 5d | IP | 1w,2w,3w |
| 55 | Li,  2009 | China | RCT | SD rats | Female and Male | 250-300g | Adult | 5/5 | Contusion | T10 | 25gcm(5g×5cm) | TMP | 200mg/kg,once per day for 5d | IP | 1w,2w |
| 56 | Song,  2009 | China | RCT | SD rats | Female | 200-250g | / | 6/6 | Contusion | T9 | 50gcm(5g×10cm) | GS | 5mg/kg,once per day for 14d | IP | 1w,2w |
| 57 | Li,  2008 | China | RCT | SD rats | Male | 250-300g | Adult | 5/5 | Contusion | T10 | 25gcm(5g×5cm) | TMP | 200mg/kg,once per day for 5d | IP | 1w,2w |
| 58 | Li,  2007 | China | RCT | SD rats | Female and Male | 200-250g | Adult | 5/5 | Contusion | T10 | 20gcm(5g×4cm) | TMP | 200mg/kg,once per day for 5d | IP | 1w,2w,3w |
| 59 | Sun,  2004 | China | RCT | SD rats | Female | 200-250g | 16w | 8/8 | Contusion | T6-10 | 25gcm(10g×2.5cm) | TMP | 200mg/kg,once per day for 6d | IP | 1w |

**Table S3: Ranking plot of motor function recovery in the first week after treatment with TCM Monomers**

| **Rank/Drugs** | **CUR** | **TMP** | **RSV** | **GS** | **TIIA** | **Placebo** |
| --- | --- | --- | --- | --- | --- | --- |
| **Rank 1** | 0.001 | 0.014 | 0.291 | 0.108 | 0.586 | 0 |
| **Rank 2** | 0.01 | 0.088 | 0.415 | 0.264 | 0.224 | 0 |
| **Rank 3** | 0.053 | 0.293 | 0.195 | 0.349 | 0.112 | 0 |
| **Rank 4** | 0.187 | 0.48 | 0.076 | 0.201 | 0.057 | 0 |
| **Rank 5** | 0.749 | 0.127 | 0.023 | 0.079 | 0.021 | 0 |
| **Rank 6** | 0 | 0 | 0 | 0 | 0 | 1 |

**Table S4: Ranking plot of motor function recovery in the second week after treatment with TCM Monomers**

| **Rank/Drugs** | **CUR** | **TMP** | **RSV** | **GS** | **TIIA** | **Placebo** |
| --- | --- | --- | --- | --- | --- | --- |
| **Rank 1** | 0.006 | 0.048 | 0.543 | 0.176 | 0.228 | 0 |
| **Rank 2** | 0.028 | 0.191 | 0.274 | 0.328 | 0.179 | 0 |
| **Rank 3** | 0.1 | 0.354 | 0.116 | 0.272 | 0.158 | 0 |
| **Rank 4** | 0.306 | 0.316 | 0.05 | 0.165 | 0.164 | 0 |
| **Rank 5** | 0.561 | 0.091 | 0.017 | 0.06 | 0.272 | 0 |
| **Rank 6** | 0 | 0 | 0 | 0 | 0 | 1 |

**Table S5: Ranking plot of motor function recovery in the third week after treatment with TCM Monomers**

| **Rank/Drugs** | **CUR** | **TMP** | **RSV** | **GS** | **TIIA** | **Placebo** |
| --- | --- | --- | --- | --- | --- | --- |
| **Rank 1** | 0.045 | 0.135 | 0.373 | 0.362 | 0.085 | 0 |
| **Rank 2** | 0.118 | 0.271 | 0.303 | 0.249 | 0.06 | 0 |
| **Rank 3** | 0.224 | 0.335 | 0.188 | 0.187 | 0.066 | 0 |
| **Rank 4** | 0.436 | 0.211 | 0.103 | 0.15 | 0.1 | 0 |
| **Rank 5** | 0.177 | 0.049 | 0.032 | 0.053 | 0.553 | 0.136 |
| **Rank 6** | 0 | 0 | 0 | 0 | 0.136 | 0.864 |

**Table S6: Ranking plot of motor function recovery in the fourth week after treatment with TCM Monomers**

| **Rank/Drugs** | **CUR** | **TMP** | **RSV** | **GS** | **TIIA** | **Placebo** |
| --- | --- | --- | --- | --- | --- | --- |
| **Rank 1** | 0.002 | 0.005 | 0.451 | 0.391 | 0.15 | 0 |
| **Rank 2** | 0.019 | 0.035 | 0.376 | 0.373 | 0.197 | 0 |
| **Rank 3** | 0.164 | 0.185 | 0.144 | 0.183 | 0.325 | 0 |
| **Rank 4** | 0.404 | 0.378 | 0.024 | 0.04 | 0.153 | 0 |
| **Rank 5** | 0.411 | 0.398 | 0.004 | 0.012 | 0.174 | 0 |
| **Rank 6** | 0 | 0 | 0 | 0 | 0 | 1 |

**References**

1. Fan X, Tao JW, Jiang SY, Deng BW, Mu XH. Effect of tetramethylpyrazine on iron metabolism after spinal cord injury in rats. Chin J Tissue Eng Res. (2023) 27:3561-3566.

2. Su XC, Wang GQ, Li JB, Ji L, Li M, Zhang YG, et al. Curcumin suppresses the inflammatory response after acute spinal cord injury by modulating NLRP3 inflammasomes. J Shanxi Med Univ. (2022) 53:719-723. doi: 10.13753/j.issn.1007-6611.2022.06.010.

3. Shen K, Wu D, Sun B, Zhu Y, Wang H, Zou W, et al. Ginsenoside Rg1 promotes astrocyte-to-neuron transdifferentiation in rat and its possible mechanism. CNS Neurosci Ther. (2023) 29:256-269. doi: 10.1111/cns.14000.

4. Zhang Z, Yang K, Mao R, Zhong D, Xu Z, Xu J, et al. Ginsenoside Rg1 inhibits oxidative stress and inflammation in rats with spinal cord injury via Nrf2/HO-1 signaling pathway. Neuroreport. (2022) 33:81-89. doi: 10.1097/WNR.0000000000001757.

5. Li W, Yao S, Li H, Meng Z, Sun X. Curcumin promotes functional recovery and inhibits neuronal apoptosis after spinal cord injury through the modulation of autophagy. J Spinal Cord Med. (2021) 44:37-45. doi: 10.1080/10790268.2019.1616147.

6. Zhao H, Mei X, Yang D, Tu G. Resveratrol inhibits inflammation after spinal cord injury via SIRT-1/NF-κB signaling pathway. Neurosci Lett. (2021) 762:136151. doi: 10.1016/j.neulet.2021.136151.

7. Chen YY, Zhou SJ, Xia YC, Xue XL. Effects of Curcumin on Inflammatory Factors in Rats with Acute Spinal Cord Injury. Asia-Pacific Trad Med. (2020) 16:20-22.

8. Zhang Y, Qi W, Wu D, Xie MC. Study on the Effects of Ligustrazine on Gene Expression of Acute Spinal Cord Injury Model Rats Based on Transcriptome Sequencing. China Pharm. (2020) 31:1327-1335.

9. Fan Y, Li Y, Huang S, Xu H, Li H, Liu B. Resveratrol-primed exosomes strongly promote the recovery of motor function in SCI rats by activating autophagy and inhibiting apoptosis via the PI3K signaling pathway. Neurosci Lett. (2020) 736:135262. doi: 10.1016/j.neulet.2020.135262.

10. Liu X, Botchway BOA, Tan X, Zhang Y, Fang M. Resveratrol treatment of spinal cord injury in rat model. Microsc Res Tech. (2019) 82:296-303. doi: 10.1002/jemt.23171.

11. Yu Y. The Biological Significance of Resveratrol on Inhibiting the Expression of YAP after Spinal Cord Injury in Rats. Dalian Med Univ. (2019).

12. Bang WS, Kim KT, Seo YJ, Cho DC, Sung JK, Kim CH. Curcumin Increase the Expression of Neural Stem/Progenitor Cells and Improves Functional Recovery after Spinal Cord Injury. J Korean Neurosurg Soc. (2018) 61:10-18. doi: 10.3340/jkns.2017.0203.003.

13. Ruzicka J, Urdzikova LM, Kloudova A, Amin AG, Vallova J, Kubinova S, et al. Anti-inflammatory compound curcumin and mesenchymal stem cells in the treatment of spinal cord injury in rats. Acta Neurobiol Exp (Wars). (2018) 78:358-374.

14. Ruzicka J, Urdzikova LM, Svobodova B, Amin AG, Karova K, Dubisova J, et al. Does combined therapy of curcumin and epigallocatechin gallate have a synergistic neuroprotective effect against spinal cord injury? Neural Regen Res. (2018) 13:119-127. doi: 10.4103/1673-5374.224379.

15. Meng HY, Shao DC, Li H, Huang XD, Yang G, Xu B, et al. Resveratrol improves neurological outcome and neuroinflammation following spinal cord injury through enhancing autophagy involving the AMPK/mTOR pathway. Mol Med Rep. (2018) 18:2237-2244. doi: 10.3892/mmr.2018.9194.

16. Wang P, Jiang L, Zhou N, Zhou H, Liu H, Zhao W, et al. Resveratrol ameliorates autophagic flux to promote functional recovery in rats after spinal cord injury. Oncotarget. (2018) 9:8427-8440. doi: 10.18632/oncotarget.23877.

17. Liu X, Gu X, Yu M, Zi Y, Yu H, Wang Y, et al. Effects of ginsenoside Rb1 on oxidative stress injury in rat spinal cords by regulating the eNOS/Nrf2/HO-1 signaling pathway. Exp Ther Med. (2018) 16:1079-1086. doi: 10.3892/etm.2018.6286.

18. Wang P, Lin C, Wu S, Huang K, Wang Y, Bao X, et al. Inhibition of Autophagy is Involved in the Protective Effects of Ginsenoside Rb1 on Spinal Cord Injury. Cell Mol Neurobiol. (2018) 38:679-690. doi: 10.1007/s10571-017-0527-8.

19. Li HB. Ginsenoside Rg1 promotes the recovery of nerve function after spinal cord injury in rats by regulating the polarization of macrophages/microglia. Nanjing Med Univ. (2018). doi: 10.27249/d.cnki.gnjyu.2018.000682.

20. Zhang XM, Ma J, Sun Y, Yu BQ, Jiao ZM, Wang D, et al. Tanshinone IIA promotes the differentiation of bone marrow mesenchymal stem cells into neuronal-like cells in a spinal cord injury model. J Transl Med. (2018) 16:193. doi: 10.1186/s12967-018-1571-y.

21. Hao Q, Liu J, Wang HW, Yang YL, Shen J, Zhao L, et al. Effects of different doses of curcumin on the recovery of tissue and hind limb function after spinal cord injury in rats. Shandong Med J. (2017) 57:11-14.

22. Hao Q, Wang HW, Yu Q, Shen J, Zhao L, Shi FF, et al. Effects of curcumin on the recovery of hind limb function after spinal cord injury in rats and its mechamism. Chin J Appl Physiol. (2017) 33:441-444.

23. Hu JZ, Wang XK, Cao Y, Li DZ, Wu TD, Zhang T, et al. Tetramethylpyrazine Facilitates Functional Recovery after Spinal Cord Injury by Inhibiting MMP2, MMP9, and Vascular Endothelial Cell Apoptosis. Curr Neurovasc Res. (2017) 14:110-116. doi: 10.2174/1567202614666170313114115

24. Chen X, Ding YL, Li XL, Zhao MM, et al. Effect of Ligustrazine on GFAP and cPLA2 expressions in rat model of acute spinal cord injury. China J Mod Med. (2017) 27:9-14.

25. Zhao H, Chen S, Gao K, Zhou Z, Wang C, Shen Z, et al. Resveratrol protects against spinal cord injury by activating autophagy and inhibiting apoptosis mediated by the SIRT1/AMPK signaling pathway. Neuroscience. (2017) 348:241-251. doi: 10.1016/j.neuroscience.2017.02.027.

26. Kim DK, Kweon KJ, Kim P, Kim HJ, Kim SS, Sohn NW, et al. Ginsenoside Rg3 Improves Recovery from Spinal Cord Injury in Rats via Suppression of Neuronal Apoptosis, Pro-Inflammatory Mediators, and Microglial Activation. Molecules. (2017) 22:122. doi: 10.3390/molecules22010122.

27. Yao NW, Lu Y, Shi LQ, Xu F, Cai XH. Neuroprotective effect of combining tanshinone IIA with low-dose methylprednisolone following acute spinal cord injury in rats. Exp Ther Med. (2017) 13:2193-2202. doi: 10.3892/etm.2017.4271.

28. Yang YL, Wang YF. The effect of curcumin on the expressions of COX-2 and iNOS in rats with acute spinal cord injury. Prog Anat Sci. (2016) 22:179-182.

29. Hu J, Cao Y, Wu T, Li D, Lu H. Micro-CT as a Tool to Investigate the Efficacy of Tetramethylpyrazine in a Rat Spinal Cord Injury Model. Spine (Phila Pa 1976). (2016) 41:1272-1278. doi: 10.1097/BRS.0000000000001546.

30. Huang JH, Cao Y, Zeng L, Wang G, Cao M, Lu HB, et al. Tetramethylpyrazine enhances functional recovery after contusion spinal cord injury by modulation of MicroRNA-21, FasL, PDCD4 and PTEN expression. Brain Res. (2016) 1648:35-45. doi: 10.1016/j.brainres.2016.07.023.

31. Su XH, Gao WS, Xue JW, Lin L. Effects of ligustrazine on ethological changes in rats with acute spinal cord injury, Hebei Med J. (2016) 38:2769-2771.

32. Xiong Y. Research on expression of GFAP after Intervention of Ligustrazine in Acute Spinal Cord Injuries. Guangxi Trad Chinese Med Univ. (2016).

33. Cong L, Chen W. Neuroprotective Effect of Ginsenoside Rd in Spinal Cord Injury Rats. Basic Clin Pharmacol Toxicol. (2016) 119:193-201. doi: 10.1111/bcpt.12562.

34. Yang YD, Yu X, Gao YS. Effects of intravenously injected tanshinone IIA on neurological function recovery of adult rat after spinal cord injury. Chin J Bone Jt. (2016) 5:412-418.

35. Li P. Resveratrol via STAT3/BMP pathway regulated astrocyte activation to facilitate recovery after spinal cord injury. Dalian Med Univ. (2016).

36. Machova Urdzikova L, Karova K, Ruzicka J, Kloudova A, Shannon C, Dubisova J, et al. The Anti-Inflammatory Compound Curcumin Enhances Locomotor and Sensory Recovery after Spinal Cord Injury in Rats by Immunomodulation. Int J Mol Sci. (2015) 17:49. doi: 10.3390/ijms17010049.

37. Hu J, Lang Y, Cao Y, Zhang T, Lu H. The Neuroprotective Effect of Tetramethylpyrazine Against Contusive Spinal Cord Injury by Activating PGC-1α in Rats. Neurochem Res. (2015) 40:1393-401. doi: 10.1007/s11064-015-1606-1.

38. Wang LL, Xi HT, Ma ZM, Cui M, Xin JY. Effect of Tetramethylpyrazine and Salvia Miltiorrhiza on Neurological Function Recovery of Rats after Spinal Cord Injury. Shanxi Tradit Chin Med. (2015) 31:53-55.

39. Zhang N, Zhao M, Sun YP. Effect and Mechanism of Tetramethylpyrazine onMotor Function after Spinal Cord Injury in Rats. J China Med Univ. (2015) 44:60-63.

40. Sun JZ, Liu XW, Guan HP, Zhang P, Liu Q, Yang J, et al. Effect of Ginsenoside Rg1 on transformation growth factor-beta and brain-derived neurotrophic factor expression in spinal cord injury rats. Chin. J Tissue Eng Res. (2015) 19:2862-2866.

41. Kim KT, Kim MJ, Cho DC, Park SH, Hwang JH, Sung JK, et al. The neuroprotective effect of treatment with curcumin in acute spinal cord injury: laboratory investigation. Neurol Med Chir (Tokyo). (2014) 54:387-94. doi: 10.2176/nmc.oa.2013-0251.

42. Yu DS, Cao Y, Mei XF, Wang YF, Fan ZK, Wang YS, et al. Curcumin improves the integrity of blood-spinal cord barrier after compressive spinal cord injury in rats. J Neurol Sci. (2014) 346:51-9. doi: 10.1016/j.jns.2014.07.056.

43. Zu J, Wang Y, Xu G, Zhuang J, Gong H, Yan J. Curcumin improves the recovery of motor function and reduces spinal cord edema in a rat acute spinal cord injury model by inhibiting the JAK/STAT signaling pathway. Acta Histochem. (2014) 116:1331-6. doi: 10.1016/j.acthis.2014.08.004.

44. Xiang X, Yuan JC, Chen F, Zhu HT, Chu WH, Lin JK. Curcumin induces endogenous neural stem cells to promote functional recovery in rats after spinal cord injury. J Army Med Univ. (2014) 36:883-887. doi: 10.16016/j.1000-5404.2014.09.017.

45. Hu JZ, Huang JH, Xiao ZM, Li JH, Li XM, Lu HB. Tetramethylpyrazine accelerates the function recovery of traumatic spinal cord in rat model by attenuating inflammation. J Neurol Sci. (2013) 324:94-9. doi: 10.1016/j.jns.2012.10.009.

46. Wang GQ. Effects of tetramethylpyrazine on the expression of GAP-43 and Survivin after spinal cord injury in rats. Cent South Univ. (2013).

47. Qi XT, Jiang DM, Zhu FC, Li CW, Huang RY. Protective effects of curcumine on acute spinal cord injury in rats and its mechanism. J Trauma Surg. (2013) 15:250-254.

48. Xiao ZM, Hu JH, Lv HB, Zhuo XL, Xu DQ, Wang SX, et al. Effect of tetramethylpyrazine on the expression of macrophage migration inhibitory factor in acute spinal cord injury in rats. J Cent South Univ, Med Sci. (2012) 37:1031-1036.

49. Yin X, Yin Y, Cao FL, Chen YF, Peng Y, Hou WG, et al. Tanshinone IIA attenuates the inflammatory response and apoptosis after traumatic injury of the spinal cord in adult rats. PLoS One. (2012) 7:e38381. doi: 10.1371/journal.pone.0038381.

50. Ormond DR, Peng H, Zeman R, Das K, Murali R, Jhanwar-Uniyal M. Recovery from spinal cord injury using naturally occurring antiinflammatory compound curcumin: laboratory investigation. J Neurosurg Spine. (2012) 16:497-503. doi: 10.3171/2012.1.SPINE11769.

51. Zou MM, Xue XS, Zhu HT, Li L, Xia YZ, Zhao TZ, et al. Effect of curcumin on recovery promotion after spinal cord in jury in rats: a pathological and behavioral study. J Army Med Univ. (2011) 33:765-770. doi: 10.16016/j.1000-5404.2011.08.005.

52. Qi W, Huang YR, Liu RZ, Pan HS, Han J. Effect of Tetramethylpyrazine Injection on Expression of NOS and IL-lβ after Acute Spinal Cord Injuries in Rats. Chin J Tradit Med Traumatol Orthop. (2011) 19:7-8+11.

53. Zhou B, Hu JZ, Lv HB, Li X. Influence of tetramethylpyrazine on the expression of neurotrophic factors in rats with acute spinal cord injury. Mod J Integr Tradit Chin West Med. (2010) 19:1058-1060.

54. Huang JJ. The ethology effect of treating acute spinal cord injury in the rat model with tetramethylpyrazine. Cent South Univ. (2010).

55. Li JH. The effect of tetramethylpyrazine on the expression of P-Selectin、neutrophil elastase after acute spinal cord injury in the rat model. Cent South Univ. (2009).

56. Song YX, Jin CY, Zeng YY, Wang B, Zhang JQ. Study on the mechanism underlying neuroprotective effect of Ginsenosides in rats after spinal cord injury. Hainan Med J. (2009) 20:6-9.

57. Li XM. The effect of tetramethylpyrazine on the expression of IL-10、IL-18 after acute spinal cord injury in the rat model. Cent South Univ. (2007).

58. Li Y. Experimental study of TMP on spinal cord injury microcirculation of rats. Cent South Univ. (2007).

59. Sun HY, Jia LS, Chen XW, Gui BJ. Tetramethylpyraze is neuroprotective after acute experimental spinal cord trauma in rats. The J Cervicodynia Lumbodynia. (2004) 6:395-398.
